# Brain Abscess Following Definitive Radiotherapy in Patients With External Auditory Canal Carcinoma: Report of Two Cases

**DOI:** 10.7759/cureus.81592

**Published:** 2025-04-01

**Authors:** Sho Iwaki, Daisuke Kawakita, Takuma Matoba, Kiyoshi Minohara, Shinichi Iwasaki

**Affiliations:** 1 Department of Otolaryngology, Head and Neck Surgery, Nagoya City University Graduate School of Medical Sciences, Nagoya, JPN

**Keywords:** brain abscess, external auditory canal, radiotherapy, squamous cell carcinoma, temporal bone osteonecrosis

## Abstract

While osteoradionecrosis of the temporal bone is a late complication of radiotherapy (RT) for external auditory canal carcinoma (EACC), brain abscesses are rare. We present two cases of EACC treated with definitive RT, both of whom subsequently developed brain abscesses. The first patient, a 65-year-old woman with right EACC invading the sigmoid sinus and dura mater, developed a cerebellar abscess eight years post-RT (60 Gy/30 Fr). This abscess, attributed to infection related to temporal bone osteoradionecrosis, was successfully managed with drainage and antibiotics. The second patient, a 45-year-old man with right EACC and suspected dura mater invasion developed a right temporal lobe abscess two months post-RT (70 Gy/35 Fr). Owing to its persistence despite drainage and antibiotics, the abscess was resected, and the skull base was reinforced with a temporalis muscle flap. Neither patient experienced local recurrence. We hypothesize that the brain abscesses resulted from the vulnerability of the dura mater and skull base. Brain abscesses should be considered a potential adverse outcome following definitive RT for EACC, particularly when tumor invasion of the dura mater or skull base is suspected.

## Introduction

External auditory canal carcinoma (EACC) is an exceedingly rare malignancy, accounting for <0.2% of all tumors and having a relatively poor prognosis [1]. In the absence of a UICC staging system, the modified Pittsburgh classification is widely adopted, relying on intraoperative findings and histopathological assessments [1]. The lack of evidence from prospective randomized trials has hindered the establishment of definitive treatment guidelines for EACC [2]. Surgical resection with adequate margins is generally preferred for clinically resectable disease. However, radical surgery requires expert surgeons owing to the tumor’s rarity and the complex anatomy of the temporal bone, which involves critical structures. Additionally, subtotal and total temporal bone resection often result in severe complications, including inevitable deafness and facial nerve palsy [3]. Recent studies suggest that definitive radiotherapy (RT) combined with chemotherapy may be an effective alternative to surgery, particularly for advanced-stage disease [4-6]. A radiation dose of up to 70 Gy is recommended, as higher doses may increase the risk of temporal bone osteoradionecrosis [7,8]. Cisplatin is commonly used as a chemotherapy agent [9], and the efficacy of combining docetaxel, cisplatin, and 5-fluorouracil with RT has also been reported [6].

Stenosis of the external auditory canal and hearing impairment are the primary long-term adverse effects of RT for EACC [6]. Additionally, osteoradionecrosis of the temporal bone is a recognized late complication, with cases reported up to 10 years post-treatment [10-12]. Conversely, brain abscesses following RT for EACC are uncommon. Here, we present two cases of EACC treated with definitive RT that subsequently developed brain abscesses. The second case is particularly novel, as the brain abscess occurred as an early complication of RT for EACC-an extremely rare event with no prior case reports. This abscess was presumed to result from skull base vulnerability due to tumor invasion and RT. The first case, in contrast, developed a brain abscess as a late complication following osteoradionecrosis, a more commonly observed outcome. We present these cases to compare and clarify the difference between early- and late-onset brain abscesses following RT for EACC.

This article was previously presented as a meeting abstract at the 19th Korea-Japan Joint Meeting of Otorhinolaryngology-Head and Neck Surgery on March 20-22, 2024.

## Case presentation

Case report

Patient data were extracted retrospectively from medical charts.

Case 1

**Figure 1:**
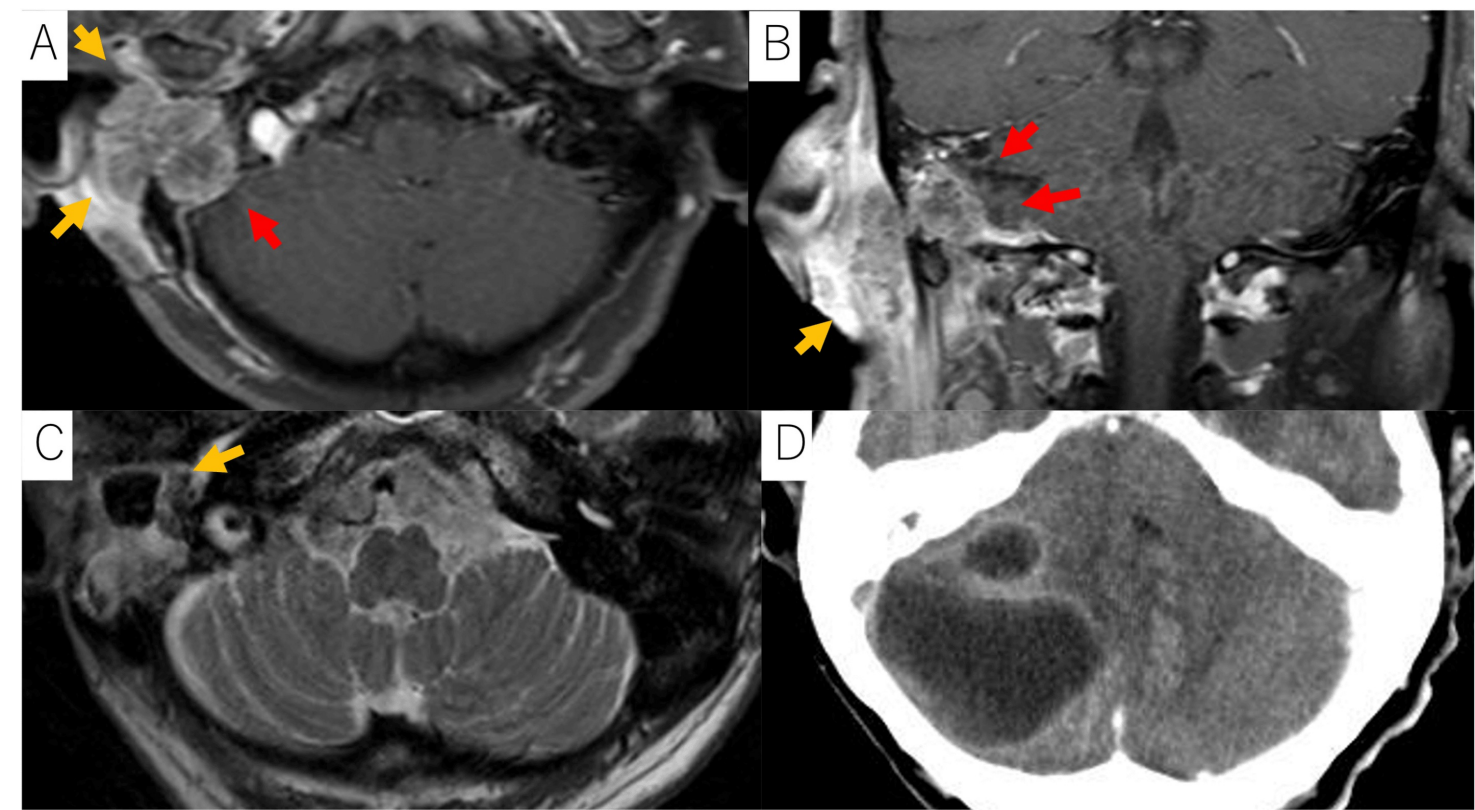
Images of the brain abscess in case 1 A, B: Magnetic resonance imaging (MRI) T1-weighted with gadolinium enhancement images demonstrating the tumor (indicated by yellow arrows and red arrows). Additionally, the image demonstrates tumor invasion into the right sigmoid sinus and dura mater (indicated by red arrows). The tumor was classified as T4 based on the modified Pittsburg classification. There was no cervical lymph node involvement or distant metastasis. C: MRI T2-weighted image reveals destruction of the right temporal bone, observed seven years post-radiation therapy (RT) (indicated by yellow arrow). D: Computed tomography (CT) scan demonstrates a ring-enhancing lesion with a low-density area in the right cerebellum, consistent with a cerebellar abscess that developed eight years post-radiotherapy. The abscess measured a maximum of 43 mm. It was managed with drainage and antibiotic therapy. The patient exhibited no evidence of recurrence or subsequent brain abscess formation.

A 65-year-old woman with a history of plaque psoriasis and acute respiratory distress syndrome presented with right external auditory canal pain. She was diagnosed with right EACC, which filled the right auditory canal and invaded the postauricular skin, right sigmoid sinus, and dura mater (Figures 1A, 1B).

**Figure 2:**
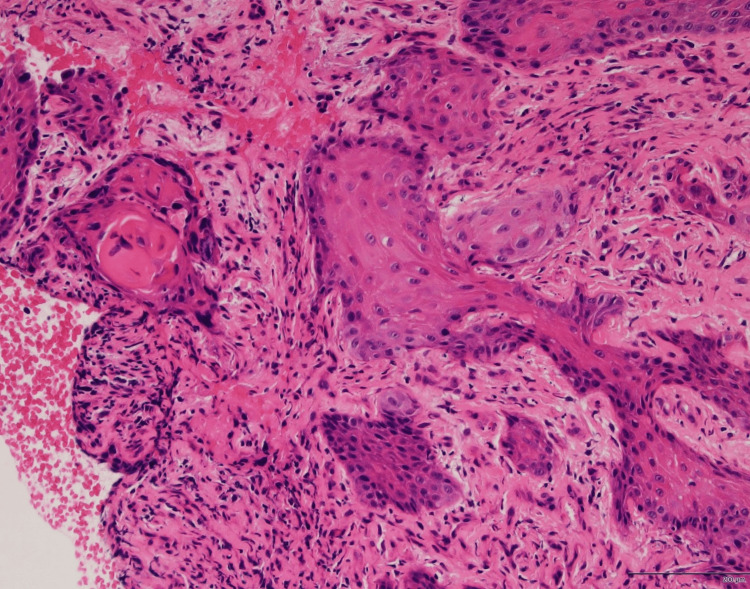
Histopathological features of the tumor in case 1 Hematoxylin and eosin staining of the specimen reveals atypical squamous epithelium invading the stroma, confirming the diagnosis of well-differentiated squamous cell carcinoma.

Histological analysis confirmed well-differentiated squamous cell carcinoma (SCC) (Figure 2), classified as T4 without cervical lymph node involvement or distant metastasis based on the modified Pittsburg classification. Positron emission tomography-computed tomography (PET-CT) also showed no evidence of cervical lymph node or distant metastasis. 

Following one cycle of induction chemotherapy with docetaxel (48 mg/m2), cisplatin (56 mg/m2), and 5-fluorouracil (480 mg/m2), the patient underwent super selective intra-arterial cisplatin therapy (first: 150 mg, second to fourth: 145 mg, weekly) and RT (60 Gy/30 Fr), resulting in a complete response. Intensity-modulated radiation therapy (IMRT) was used to target the primary tumor. 

Shortly after RT, the patient developed persistent otorrhea in the right ear. Four years post-RT, she also developed a chronic postauricular skin fistula. Regular follow-up with contrast-enhanced magnetic resonance imaging (MRI) and PET-CT was conducted to monitor disease progression and treatment effects. Seven years after initial treatment, an MRI revealed destruction of the right temporal bone, suggestive of osteoradionecrosis (Figure 1C). Eight years post-treatment, the patient developed fever and vomiting. A CT scan demonstrated a ring-enhancing lesion with a low-density area in the right cerebellum, consistent with a cerebellar abscess measuring 43 mm at its maximum diameter (Figure 1D). MRI T1-weighted imaging demonstrated low signal intensity, while T2-weighted imaging revealed high signal intensity. Laboratory tests indicated elevated white blood cell counts (11800/µL) and an increased C-reactive protein level (1.98 mg/dL). The abscess was managed with drainage and antibiotic therapy. *Enterococcus avium*, *Corynebacterium sp.*, *Pseudomonas aeruginosa*, *Paenibacillus timonesis*, and *Bacteroides fragilis* were identified within the abscesses. Ceftriaxone, metronidazole, and linezolid were administered.

The patient had a chronic external auditory canal infection with persistent otorrhea, which started shortly after RT and persisted throughout follow-up. Over the years, a postauricular skin fistula and osteoradionecrosis of the temporal bone developed. We hypothesize that chronic infection spreads intracranially through the skin fistula and the osteoradionecrotic temporal bone, ultimately leading to the brain abscess. The patient exhibited no evidence of recurrence or subsequent brain abscess formation.

Case 2

**Figure 3:**
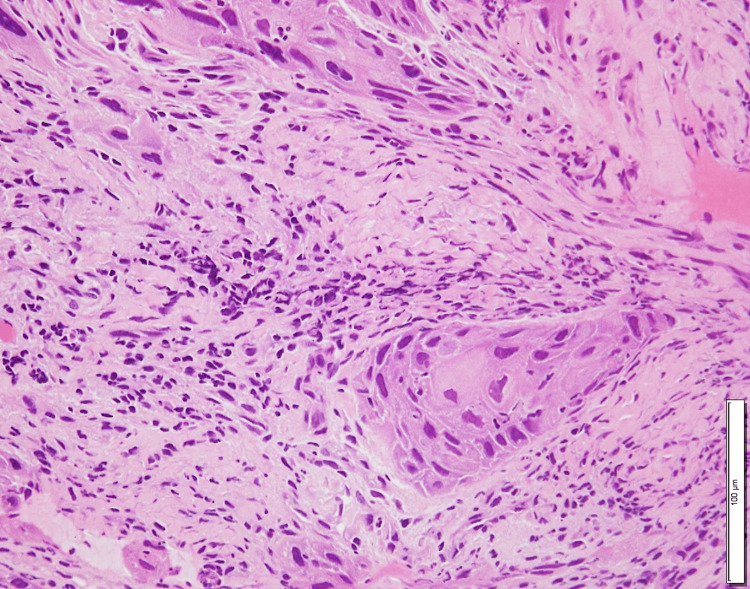
Histopathological features of the tumor in case 2 Hematoxylin and eosin staining of the specimen reveals atypical squamous epithelium with stromal invasion, confirming the diagnosis of moderately differentiated squamous cell carcinoma.

A 45-year-old man with a history of anti-NMDAR encephalitis presented with a right EACC involving the entire external auditory canal. His chief complaint was right-sided otorrhea. The patient was not immunocompromised. Histopathological analysis confirmed a diagnosis of moderately differentiated SCC (Figure 3).

**Figure 4:**
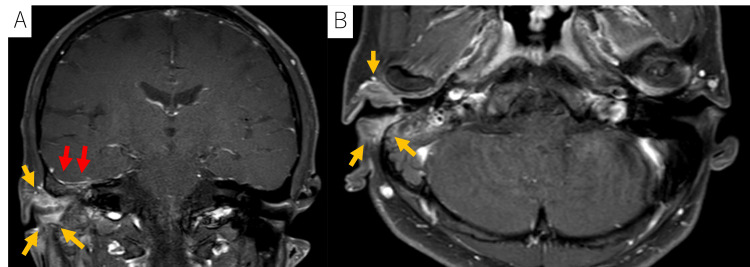
Images of the tumor in case 2 A, B: MRI images revealing the tumor (indicated by yellow arrows) and enhancement of the dura mater in the right middle cranial fossa (indicated by red arrows). The disease was classified as T4 based on the modified Pittsburgh classification. The patient had no cervical lymph node involvement or distant metastasis.

MRI demonstrated enhancement of the right middle cranial fossa dura mater, indicative of tumor invasion (Figures 4A, 4B). The disease was classified as T4 based on the modified Pittsburgh classification. There was no evidence of cervical lymph node involvement or distant metastasis. The patient underwent definitive concurrent chemoradiotherapy with IMRT (70 Gy/35 Fr) with cisplatin (100 mg/m2, three triweekly courses).

**Figure 5:**
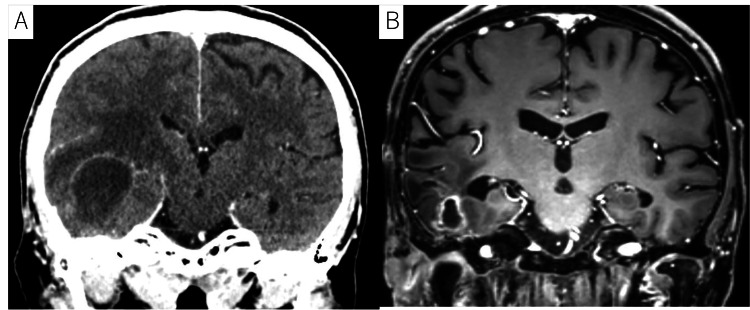
Images of the brain abscess in case 2 A: CT scan demonstrating a ring-enhancing lesion with a low-density area in the right temporal lobe, consistent with a temporal lobe abscess, measuring a maximum of 36 mm. The brain abscess developed two months after RT. B: The brain abscess persisted despite drainage and antibiotic treatment.

Two months post-treatment, the patient was transported to the emergency department with impaired consciousness as the chief complaint. A CT scan demonstrated a ring-enhancing lesion with a low-density area in the right temporal lobe, consistent with a temporal lobe abscess measuring 36 mm at its maximum diameter (Figure 5A). Immediate drainage and antibiotic therapy were initiated. *Campylobacter ureolyticus* was identified in both the abscesses and otorrhea, suggesting that the infection originated from the external auditory canal. Initial antibiotic treatment with meropenem and linezolid was adjusted to ceftriaxone and metronidazole based on bacterial susceptibility. Despite antibiotic therapy, the abscess persisted, with ongoing signs of active infection (Figure 5B).

**Figure 6:**
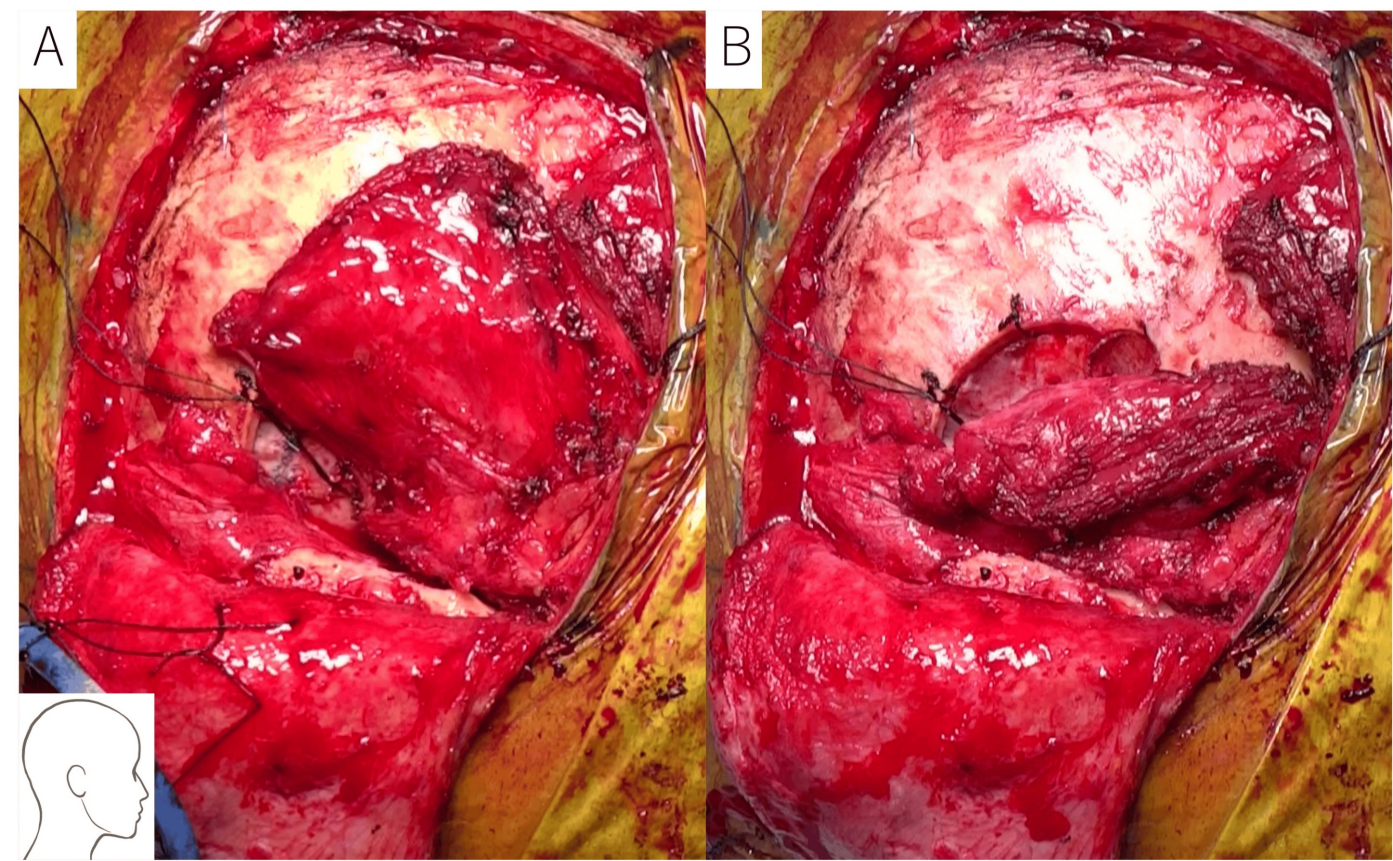
Pictures of the pedicled temporalis muscle flap created in case 2 To treat the abscess, a partial temporal lobectomy was performed. A: A pedicled temporalis muscle flap was created following resection of the brain abscess. B: The flap was positioned between the dura mater and the skull base. The patient remains alive with no recurrence or subsequent brain abscess formation.

To achieve definitive treatment, a partial temporal lobectomy was performed. Temporal bone resection and free-flap transplantation were considered if osteonecrosis was found. Intraoperatively, no osteonecrosis or recurrence was detected. A small fistula connecting the mastoid cells to the middle cranial fossa was identified, necessitating skull base reinforcement with a pedicled temporalis muscle flap (Figures 6A, 6B). Pathological examination revealed a cancer pearl within the abscess, suggesting SCC invasion of the skull base, although viable tumor cells were absent. The brain abscess was attributed to the compromised dura mater and skull base resulting from tumor invasion and RT. The patient remains alive without recurrence or subsequent brain abscesses.

## Discussion

We encountered two cases of brain abscesses following definitive RT for EACC. The first patient developed a brain abscess as a late complication due to osteoradionecrosis. The second case represented an atypical early-onset brain abscess after RT for EACC.

Brain abscesses post-RT for EACC are exceedingly rare, with only a few cases reported in the literature. Fang et al. and Chuang et al. described brain abscesses occurring after RT for nasopharyngeal carcinoma [10,11], but no similar case series exists for EACC. This scarcity likely reflects the rarity of EACC and the availability of both surgical and RT options compared to nasopharyngeal carcinoma. Laton reported a case of middle ear SCC treated with surgery and postoperative RT, resulting in temporal bone osteoradionecrosis and cerebellar abscess, akin to our first case [13]. However, unlike our second case, there are no reports of early-onset brain abscesses without concurrent osteoradionecrosis.

The complex anatomy of the temporal bone and skull base influences disease progression. Leonetti et al. categorized advanced temporal bone malignancies into five invasion patterns: (a) superior erosion through the tegmen tympani into the middle cranial fossa, (b) anterior extension into the glenoid fossa and infratemporal space, (c) inferior growth through the hypotympanum and jugular foramen, (d) posterior involvement of the mastoid air cells, and (e) medial involvement of the middle ear and carotid canal [14]. Our first case aligned with pattern (d), primarily involving the mastoid cell, while the second case resembled pattern (a), predominantly affecting the middle cranial fossa. Given this, brain abscesses should be considered a potential sequela of RT in cases with skull base invasion.

Nevertheless, not all patients with skull base invasion develop post-RT brain abscesses. Yamada et al. reported no brain abscesses in patients with EACC with brain invasion treated with RT and chemotherapy (docetaxel, cisplatin, or 5-fluorouracil) [5]. Meanwhile, our second case suggests that factors beyond skull base invasion contribute to brain abscess formation. The method of radiation delivery, chemotherapy regimen, mode of SCC invasion, and patient-specific characteristics may influence skull base vulnerability. However, owing to the absence of reports on early-onset brain abscesses following RT for EACC, we could not confirm a definitive causative factor. Further studies and case reports are needed to clarify this issue.

Regarding osteoradionecrosis, Ramsden et al. classified the condition into localized and diffuse forms [7]. Conservative management is often suitable for localized disease, whereas surgical intervention is usually required for diffuse disease. Chen et al. compared treatment outcomes for mastoid osteoradionecrosis post-RT for nasopharyngeal cancer, finding that the temporalis muscle flap is superior to mastoidectomy in preventing purulent otorrhea and persistent osteoradionecrosis [15]. Although our second case did not necessitate lateral bone resection, we reinformed the skull base with a pedicled temporalis muscle flap owing to the presence of a middle cranial fossa fistula. No recurrent brain abscesses have occurred postoperatively.

This case report includes only two cases, and there are only a few cases that report the occurrence of brain abscesses post-RT for EACC. Therefore, it is difficult to comprehensively discuss the mechanism of this issue. For a deeper understanding, more case reports on this condition are warranted.

## Conclusions

We encountered one case each of early- and late-onset brain abscess following definitive RT for EACC. Brain abscesses should be considered as potential adverse events at both early and late stages post-RT for EACC when tumor invasion of the dura mater or skull base is suspected.
